# Genetic Variants in *CHIA* and *CHI3L1* Are Associated with the IgE Response to the *Ascaris* Resistance Marker ABA-1 and the Birch Pollen Allergen Bet v 1

**DOI:** 10.1371/journal.pone.0167453

**Published:** 2016-12-15

**Authors:** Nathalie Acevedo, Adriana Bornacelly, Dilia Mercado, Per Unneberg, Irene Mittermann, Rudolf Valenta, Malcolm Kennedy, Annika Scheynius, Luis Caraballo

**Affiliations:** 1 Institute for Immunological Research, University of Cartagena, Cartagena, Colombia; 2 Science for Life Laboratory, Department of Clinical Science and Education, Karolinska Institutet, and Sachs' Children and Youth Hospital, Södersjukhuset, Stockholm, Sweden; 3 Science for Life Laboratory, Department of Biochemistry and Biophysics, Stockholm University, Solna, Sweden; 4 Department of Pathophysiology and Allergy Research, Division of Immunopathology, Center for Pathophysiology, Infectology and Immunology, Medical University of Vienna, Vienna, Austria; 5 College of Medical, Veterinary and Life Sciences, University of Glasgow, Glasgow, Scotland, United Kingdom; Central University of Tamil Nadu, INDIA

## Abstract

Helminth infections and allergic diseases are associated with IgE hyperresponsiveness but the genetics of this phenotype remain to be defined. Susceptibility to *Ascaris lumbricoides* infection and antibody levels to this helminth are associated with polymorphisms in locus 13q33-34. We aimed to explore this and other genomic regions to identify genetic variants associated with the IgE responsiveness in humans. Forty-eight subjects from Cartagena, Colombia, with extreme values of specific IgE to *Ascaris* and ABA-1, a resistance marker of this nematode, were selected for targeted resequencing. Burden analyses were done comparing extreme groups for IgE values. One-hundred one SNPs were genotyped in 1258 individuals of two well-characterized populations from Colombia and Sweden. Two low-frequency coding variants in the gene encoding the Acidic Mammalian Chitinase (*CHIA* rs79500525, rs139812869, tagged by rs10494133) were found enriched in high IgE responders to ABA-1 and confirmed by genetic association analyses. The SNP rs4950928 in the Chitinase 3 Like 1 gene (*CHI3L1*) was associated with high IgE to ABA-1 in Colombians and with high IgE to Bet v 1 in the Swedish population. *CHIA* rs10494133 and *ABDH13* rs3783118 were associated with IgE responses to *Ascaris*. SNPs in the Tumor Necrosis Factor Superfamily Member 13b gene (*TNFSF13B*) encoding the cytokine B cell activating Factor were associated with high levels of total IgE in both populations. This is the first report on the association between low-frequency and common variants in the chitinases-related genes *CHIA* and *CHI3L1* with the intensity of specific IgE to ABA-1 in a population naturally exposed to *Ascaris* and with Bet v 1 in a Swedish population. Our results add new information about the genetic influences of human IgE responsiveness; since the genes encode for enzymes involved in the immune response to parasitic infections, they could be helpful for understanding helminth immunity and allergic responses. We also confirmed that *TNFSF13B* has an important and conserved role in the regulation of total IgE levels, which supports potential evolutionary links between helminth immunity and allergic response.

## Introduction

Upon infection with helminths humans synthesize specific IgE antibodies to parasite components as well as high levels of total IgE. The intensity of this response differs among exposed individuals [1–4], which seems to be determined by environment and their genetic backgrounds. Studies in animals [5–7] suggest that the specificity of the IgE to helminth components is determined by alleles of the major histocompatibility complex (MHC). However, the complete set of genes regulating this response and to common allergens is not defined.

The intestinal helminth *Ascaris lumbricoides* infects about 0.9 billion people worldwide [8], inducing specific IgE against its proteins (e.g. the polyprotein allergen ABA-1, tropomyosin, glutathione-S-transferase) [9–11], high levels of total IgE and, in general, a strong Th2 response [12]. Therefore, the increase in IgE elicited by *Ascaris* infection (ascariasis) is a good model for analyzing the genetics of IgE responsiveness. In addition, ABA-1 (also designated Asc s 1) is considered a resistance marker for *Ascaris* and also an *Ascaris*-specific component since it has no cross-reactivity with house dust mite (HDM) allergens [13, 14]. ABA-1 is therefore an excellent tool with which to investigate the genetics of the IgE response to *Ascaris*.

Genetic studies on the susceptibility to *Ascaris* have identified several associated loci including the signal transducer and activator of transcription 6 (*STAT6*) [15, 16], β2-adrenoreceptor (*ADRB2*) [17], tumor necrosis factor superfamily member 13B (*TNFSF13B*) [18–20], and ligase 4 (*LIG4*) [20]. Still, very little is known about the genetic influences on the IgE response to *Ascaris* and *Ascaris*-specific components such as ABA-1 in humans. Also, it remains to be defined whether the IgE responses to helminth and environmental allergens are under the same genetic control, which is of evolutionary significance given the features that anti-helminth immunity and allergic inflammation have in common [21, 22]. This question has been addressed by genetic epidemiology studies [15, 16, 23] and bioinformatics approaches [24] that evaluate each phenotype in separate populations, but studying the problem in populations naturally exposed to both helminths and allergens could be more informative.

We previously observed a great inter-individual variation in the IgE antibody response to *Ascaris* [1, 4, 25] that allowed the grouping of individuals into “high" and "low" IgE response phenotypes. In preliminary studies using few tag-SNPs, we detected associations between polymorphisms in chromosome 13q33 and IgE levels to *Ascaris* [20]. In the present work we fine-mapped these signals and explored other genes that might be of relevance, under the hypothesis that total and specific IgE levels are complex traits influenced by combinations of common and rare variants. To explore if these variants may also affect the IgE responses to non-parasite allergens we included a sample set of Swedish allergic patients. The aims of this study were (1) to perform targeted resequencing of promoters, untranslated regions (UTR), exons, and introns of 14 genomic regions to identify genetic variants associated with IgE responsiveness to *Ascaris*, (2) from these variants to select a panel for genotyping two populations with different genetic and environmental backgrounds and (3) to investigate whether variants influencing IgE response to Ascaris are associated with the IgE response to non-parasite allergens. We here detected significant associations between polymorphisms in chitinase related genes and the intensity of the specific IgE response to the *Ascaris* resistance marker ABA-1 supporting that genetic factors play an important role in host responses to this parasite. We also add evidence suggesting that genes at 13q.33 locus are involved in the regulation of total and specific IgE response in humans.

## Materials & Methods

### Ethics statement

This study was conducted following the ethical principles for medical research stated in the Declaration of Helsinki. The Bioethics Committee of the University of Cartagena (Res. 26/06/2009) and the Swedish Regional Ethics Committee (Drn. 2011/1051-31) approved the study. Written informed consent was obtained from all subjects. Parents/guardians provided informed consent on behalf of all child participants.

### Population characteristics and samples

For resequencing phase, forty eight subjects from Cartagena, Colombia (CGA cohort, see below) at the extremes of the distribution (≤ 25^th^ and ≥75^th^ percentiles) of specific IgE levels to *Ascaris* and ABA-1 were included **(Table 1).** For the genotyping phase, samples from 1258 individuals from two independent cohorts (Colombia and Sweden) were selected to analyze genetic associations with total serum IgE, specific IgE to *Ascaris*, ABA-1 and other non-parasitic allergenic sources.

**Table 1:** Descriptive of individuals selected for targeted re-sequencing from the Candidate Genes for Asthma (CGA) cohort

Variables	low IgE (n = 20)	high IgE (n = 28)	*p* value^e^
Age, years (mean ±SD)	46.9 ± 17.1	34.8 ± 20.6	0.03
Gender, female (%)	9 (45)	16 (57)	0.4
Asthma, n (%)^a^	10 (50)	14 (50)	1
Total IgE (IU/ml)^b^	239 (145–826)	807 (447–1145)	0.01
IgE to Ascaris (OD)^b^	0.08 (0.07–0.08)	0.27 (0.23–0.52)	<0.001
IgE to ABA-1 (OD)^b^^,^ ^c^	0.08 (0.08–0.08)	0.48 (0.34–0.72)	<0.001
IgG to Ascaris (OD)^b^^,^ ^d^	2.04 (1.31–2.58)	2.77 (2.31–3.03)	0.003

^a^ Asthmatic patients are a representative group for analyzing IgE response to *Ascaris* since it has been described that asthmatics have a higher antibody response to nematodes [1, 20].

^b^Median (interquartile range).

^c^ABA-1 is a fatty acid binding protein of 14.6 kD, very abundant in the pseudocelomic fluid of adult parasites and considered a resistance marker to *Ascaris* infection [13, 14].

^d^ Levels of IgG to *Ascaris* extract denote that individuals in both groups have been exposed to the parasite.

^e^ Comparisons of continuous variables calculated by *t*-test (age) and Mann Whitney U test (IgE variables); and by chi square for categorical variables. IU, international units; OD, optical density units.

In both cohorts, individuals with allergic diseases (i.e. asthma, eczema) were included to provide more subjects with specific allergen IgE sensitization and to model the effect of genotypes on total IgE levels.

The Colombian cohort (Candidate Genes for Asthma, CGA) consists of 988 subjects; 597 non-asthmatic controls and 391 asthmatics **(Table 2).** Asthma was defined according to the Global Initiative for Asthma criteria using a standardized questionnaire previously tested in patients with a history of physician-diagnosed asthma. A physician belonging to the research staff confirmed the diagnosis. Subjects meeting the following criteria were recruited: current asthma, ≥8 years old and a history of ≥2 years of asthma, ≥3 episodes of asthma symptoms (wheezing, chest tightness and dyspnea) in the last 12 months or absence of symptoms due to the use of antiasthmatic medications. Children under 8 years of age were excluded to avoid asthma misdiagnosis due to the high prevalence of transitory wheezing in this age range. Unrelated control subjects without a history of asthma, allergy or other diseases were recruited randomly from the same neighborhoods as the patients, using a questionnaire. All participants lived in an urban, non -industrialized setting, belonging to the lower three (out of six) socio-economic strata in the city, where most people are naturally exposed to HDM [26] and *A*. *lumbricoides* and receive periodically anthelminthic treatment. The genetic background of this population resulted from racial admixture between Native Americans, Spaniards, and an important proportion (37.9%) of African ancestry [27, 28]. The DNA samples (from peripheral blood) were obtained from a well-characterized repository at the Institute for Immunological Research in Cartagena, Colombia; they were extracted between 2002 and 2004 and have been kept at -80°C [1, 20]. Each DNA sample used for sequencing was evaluated for DNA integrity by visualization in 1% agarose gel and had A_260_/A_280_ ratio between 1.8 and 2.09 (mean, sd, 1.93±0.05). Both cases and controls had total IgE and specific IgE to Ascaris and HDM and statistical analyses were adjusted by disease status.

**Table 2:** Descriptive of the two populations analyzed in genetic association tests

*Candidate Genes for Asthma cohort (CGA*, *n = 988)*			
**Variables**	**Non-asthmatics controls (n = 597)**	**Asthmatic patients (n = 391)**	***p* value**^**c**^
Age years (mean ± SD)	35.6 ±18	36.1 ±18.1	0.6
Gender, female (%)	339 (56.8)	246 (63)	0.06
Total IgE (IU/ml)^a^^,^^b^	125.4 (46.9–297.8)	714.8 (250–1074.5)	<0.001
Ig levels to parasite (OD)^a^			
IgE to Ascaris	0.105 (0.091–0.132)	0.118 (0.101–0.154)	<0.001
IgE to ABA-1	0.119 (0.099–0.157)	0.122 (0.098–0.187)	0.1
IgG to Ascaris	2.11 (1.63–2.62)	2.01 (1.63–2.39)	0.02
IgE levels to HDM (OD)^a^^,^^b^			
IgE to *D*. *pteronyssinus*	0.097 (0.088–0.119)	0.209 (0.117–0.605)	<0.001
IgE to *B*. *tropicalis*	0.098 (0.088–0.120)	0.279 (0.114–1.30)	<0.001
*Swedish Eczema Study cohort (n = 270)*			
**Variables**	**Healthy controls (n = 100)**	**Eczema patients (n = 170)**	***p* value**^**c**^
Age years (mean ±SD)	37.6 ± 14.3	33 ± 13.6	0.008
Gender, female (%)	61 (61)	102 (60)	0.8
Objective SCORAD index	0	33 (27–41)	-
Asthma and/or rhinitis, n (%)	0	133 (78.2)	-
Phadiatop positive, n (%)	10 (10)	129 (75.8)	<0.001
Total serum IgE (kU/l)^a^	21.5 (13–46.5)	160 (51.2–852.5)	<0.001
Fel d 1 IgE (ISU)^a^	nd	0.30 (0–4.5)	-
Bet v 1 IgE (ISU)^a^	nd	0.30 (0–10.3)	-

^a^ Median (interquartile range)

^b^ Data for 389 asthmatics and 593 non-asthmatics controls

^c^ Comparisons of continuous variables calculated by *t*-test (age) and Mann Whitney U test (antibody variables); and by chi-square for gender.

IU, international units; OD, optical density units; HDM: house dust mites

ISU: ISAC standardized units; SCORAD (SCORing Atopic Dermatitis) index: A clinical tool used to assess the extent and severity of eczema; nd = not determined.

The Swedish cohort (Swedish Eczema Study) comprised 170 atopic-dermatitis (AD) patients and 100 healthy controls. They were recruited from the Stockholm area and examined by a dermatologist at the Dermatology and Venereology Unit, Karolinska University Hospital in Stockholm, Sweden, during September until May to avoid the summer season as previously described [29, 30]. Inclusion criteria for AD patients were: diagnosis according to the UK working party, moderate to severe eczema, and skin lesions not only restricted to the hands. The severity of the eczema was assessed using the objective SCORAD index. The healthy controls were subjects who did not have clinical symptoms or history of allergy or skin disease and were genotyped to serve as controls in the estimation of allele frequencies in this population. DNA samples were extracted from peripheral blood using the QIAamp DNA Blood Mini Kit according to the manufacturer’s instructions (QIAGEN, Hilden, Germany). The demographical characteristics of this population are presented in **Table 2.** We assumed that there is no exposure to *Ascaris* in this cohort, therefore it was not tested for *Ascaris* allergens and the analyses on the IgE responses to *Ascaris* and ABA-1 were done in the CGA cohort.

### Allergens and IgE determinations

In the CGA cohort, total IgE was determined in duplicate using an enzyme-linked immunosorbent assay (ELISA) kit (RIDASCREEN; R-Biopharm, Darmstadt, Germany) according to the manufacturer’s instructions. Specific IgE to *Ascaris* extract and ABA-1 (bacterial recombinant type 1A unit of the As-NPA array of the polyprotein [31, 32] as well as specific IgE to HDM extracts (*Blomia tropicalis* and *Dermatophagoides pteronyssinus*) were detected by ELISA as described previously [20]. In the Swedish cohort, total IgE and specific IgE to any of 11 common aeroallergen sources (Phadiatop®) were measured in plasma using ImmunoCAP™ (Phadia AB, Uppsala, Sweden). Specific IgE to the purified recombinant allergens Fel d 1 (from cat) and Bet v 1 (birch pollen) were analyzed with the customized MeDALL allergen-chip (Phadia Multiplexing, Thermo Fisher Scientific, Vienna, Austria) as described by Lupinek *et al* [33]. In brief, sera samples were tested undiluted, and after washing and rinsing, arrays were scanned by a confocal laser scanner and evaluated by the Microarray Image Analyzer v3..1.2 software (Phadia AB). ISAC standardized units (ISU) of IgE reactivity to Bet v 1 and Fel d 1 were used for quantitative trait analyses because these allergens were the most frequent sensitizers in this population [30] and the distribution of IgE levels allowed modeling the effect of genetic variants.

### Targeted resequencing

Targeted resequencing was performed in 14 genes (*CHIA*, *CHI3L1*, *FCER1A*, *IL10*, *TSLP*, *IL5*, *RAD50/IL13*, *IL4*, *IL33*, *STAT6*, *LIG4*, *ABHD13*, *TNFSF13B* and *IRS2*) to evaluate their genetic variation and select markers of IgE hyper-responsiveness for further association studies.

Genomic coordinates of the coding and non-coding regions of the 14 genes included in the study were extracted via the UCSC browser. A library of RNA baits (120 mer) was designed using e-Array and produced by chemical synthesis (Agilent Technologies). For targeted enrichment, 3 μg genomic DNA from each individual was fragmented by sonication (Covaris S2 instrument) and then linked with specific adaptors and indexes. The samples were incubated overnight with the biotinylated RNA baits (SureSelect, Agilent). After targeted selection using magnetic streptavidin beads, the enriched regions were eluted according to the manufacturer's instructions. After amplification, the samples were sequenced using a 100 bp sequencing protocol (paired-end). The sequencing runs were performed according to manufacturer’s instructions (Illumina) with a setup aiming for a minimum coverage of 30X in the targeted regions. The production of the libraries and the sequencing procedures were done at Science for Life Laboratory in Stockholm, Sweden. The flow chart for data analysis is presented in **S1 Fig.**

### Data processing

Sequence reads passing Ilumina’s chastity filter were aligned to human genome reference version 19 (hg19) and post-processed for variant calling. Read alignment was done for each sample with BWA version 0.6.2 [34] and sample-specific bam files were generated (Sequence Alignment Map-format in binary format). We used Picard tools version 1.126 to sort the bam files, mark duplicates, and calculate alignment, insertion and hybrid selection metrics. Genome Analysis Tool Kit (GATK) [35] version 3.3–0 base quality recalibration (BQR) and local realignment were applied around indels. Variant calling, filtering, and variant quality score recalibration (VQSR) was done following best practice guidelines [36]. Alignments and variants were visualized with the Integrative Genome Viewer (IGV) version 2.3.19 [37]. Variants were annotated with ANNOVAR [38] and SnpEff [39]. Phasing information for each gene was obtained by running GATK Read Backed Phasing on reads mapping to target gene regions including 50 kb flanking regions. Finally, 2423 raw variants that passed filtering criteria were uploaded and analyzed into Ingenuity Variant Analysis software (www.qiagen.com/ingenuity) from QIAGEN Redwood City.

### Burden analysis

The.vcf files containing all the variants per individual were uploaded to the Ingenuity Variant Analysis software. The burden of variants according to specific IgE levels to *Ascaris* and ABA-1 was calculated between groups of high IgE responders (HR, levels ≥ percentile 75^th^) and low IgE responders (LR, levels ≤ percentile 25^th^). Ingenuity Variant Analysis software identifies genes that exhibit significant differences in variants of low frequency (MAF < 0.05) between groups. The statistical test is based on an extension of the Optimal unified Sequence Kernel Association Test (SKAT-O) and can be used to find variants associated with dichotomous and quantitative traits [40]. Starting from 2423 variants, we used the filter “confidence” to select those with a high call quality (CQ) and read depth (RD), and this resulted in 1955 variants for analysis (CQ = 100 and RD = 30). To identify variants with putative functional effects, the predicted deleterious filter was also applied. After this filter 338 variants remained in the analysis, with a predicted effect or association with a phenotype according to the American College of Medical Genetics and Genomics guidelines; and/or association with gain or loss function of a gene. The genetic analysis was done at three levels: gene-gene, variant-variant and gene-variant, including variants that occur in at least 4 high responders but not in low responders. The statistical association analysis was done for binary (HR, LR) and the specific IgE levels to *Ascaris* and ABA-1. Variants with a *p* value less than 0.05 after Bonferroni corrections and with an odds ratio greater than or equal to 1.5 between high and low responders were considered statistically significant. In addition, for quantitative traits the analysis was corrected by age, gender and asthma status.

### Selection of variants and genotyping

One hundred-one SNPs were selected for genotyping by matrix-assisted laser desorption/ionization-time of flight (MALDI-TOF) mass spectrometry (SEQUENOM®, Inc.). A detailed list of SNPs genotyped is presented in **S1 Table**. The variants for genotyping were selected based on the following criteria: (1) A statistically significant association in the variants burden analysis with specific IgE levels to *Ascaris* and ABA-1; (2) the most informative TagSNPs around the regions associated in the burden analysis (3) SNPs with a clinical or functional association with total IgE levels in PubMed and dbSNP (NCBI); (4) SNPs predicted to affect transcription factor-binding sites by different publicly available bioinformatics tools (i.e. F-SNP and Genomatix software suite v 3.1) and/or (5) related to significant promoter or enhancer chromatin state annotations based on ENCODE data (explored using Haploreg2). Ninety SNPs passed the quality criteria and were further analyzed in both populations. Primer for multiplex PCR and extension reactions were designed by the SpectroDesigner software (Sequenom GmbH, San Diego, CA, USA, available on request). PCR and extension reactions were performed according to manufacturer’s standard protocols. Concordance analysis with HapMap data was performed. Ten novel variants that were found enriched in high IgE responders in the burden analysis were included in the multiplex PCR reactions (of which seven were successful). The required 50 bp upstream and downstream flanking regions were extracted from the UCSC Genome Browser using Galaxy utilities (http://galaxyproject.org/).

### Statistical analysis

The genetic association analyses between the genotyped variants (n = 91) with the risk of being a high responder (HR) (≥ percentile 75^th^) were done in PLINK v 1.09 (http://pngu.mgh.harvard.edu/~purcell/plink/). IgE levels according to genotypes were compared using non-parametric tests (Mann Whitney and Kruskall Wallis). The associations with IgE levels as a continuous variable were modeled by using median regression and quantile regression with the package (quantreg) implemented in R. The regression models were adjusted by age, gender and clinical condition (asthma in CGA and atopic eczema in Swedish cohort) considering the confounding effect of these covariates on IgE levels (p_adj_). The significant level was set at *p* < 0.05.

## Results

### Differential distribution of variants in targeted genes

Targeted resequencing in 48 individuals from the CGA cohort resulted in 2423 variants (1851 located on the targeted genes). Of these, 1290 were already known (dbSNP137) and 561 novel (without a reference SNP ID number in dbSNP build 137) including 1663 single nucleotide substitutions and 188 indels. A summary of targeted resequencing metrics by gene region is presented in **S2 Table**. The highest numbers of variants were found in *IRS2* (n = 341) and *IL-33* (n = 311) and the lowest in *IL-5* (n = 17). There were remarkable differences in the distribution of the 75 coding variants among the loci studied, suggesting different degrees of conservation. For instance, the gene *CHIA* encoding for the acidic mammalian chitinase (AMCase) contained the highest number of exonic variants with 14 non-synonymous, 6 synonymous, 1 stop-gain and 1 frameshift, while the genes encoding the cytokines *IL-10* and *TSLP* had no coding variants. Based on the ratio of the number of observed variants to those expected from the gene size, the most polymorphic genes were *LIG4*, *IRS2*, *IL13* and *CHI3L1* **(Table 3).**

**Table 3:** Distribution of variants in the genes analyzed by targeted resequencing (CGA cohort)

locus	Gene symbol	Gene name	# Variants (total)	# Novel variants	# Coding variants	Gene size (kb)	# Variants F/E (1kb)*
1p:13.2	*CHIA*	Acidic mammalian chitinase	196	27	22	29.7	6.5
1p:22.2	*IL10*	Interleukin 10	33	5	0	4.8	6.7
1q:23.2	*FCER1A*	Fc fragment of IgE, high affinity I, receptor for; alpha polypeptide	107	30	2	18.5	5.7
1q:31.1	*CHI3L1*	Chitinase 3–like 1	80	11	8	7.8	10.1
5q:22.1	*TSLP*	Thymic stromal lymphopoietin	43	10	0	6.3	6.7
5q:31.1	*IL5*	Interleukin 5	17	0	2	2.0	8.1
5q:31.1	*IL13*	Interleukin 13	30	4	1	2.9	10.2
5q:31.1	*IL4*	Interleukin 4	84	27	3	8.6	9.6
9p:24.1	*IL33*	Interleukin 33	311	98	3	42.1	7.3
12q:13.3	*STAT6*	Signal transducer and activator of transcription 6	132	71	3	16.0	8.2
13q33.3	*LIG4*	Ligase 4	179	97	9	10.9	16.3
13q33.3	*ABHD13*	AB hydrolase domain containing protein 13	87	15	1	15.8	5.4
13q33.3	*TNFSF13B*	B-cell activating factor	211	67	4	38.8	5.4
13q34	*IRS2*	Insulin receptor substrate 2	341	99	17	32.7	10.4
			1851	561	75	-	-

*The ratios of the number of variants found/expected (F/E) were calculated based on the frequency of SNP throughout the human genome, one in every 1000 base pairs.

### Burden analysis of variants

**Fig 1:**
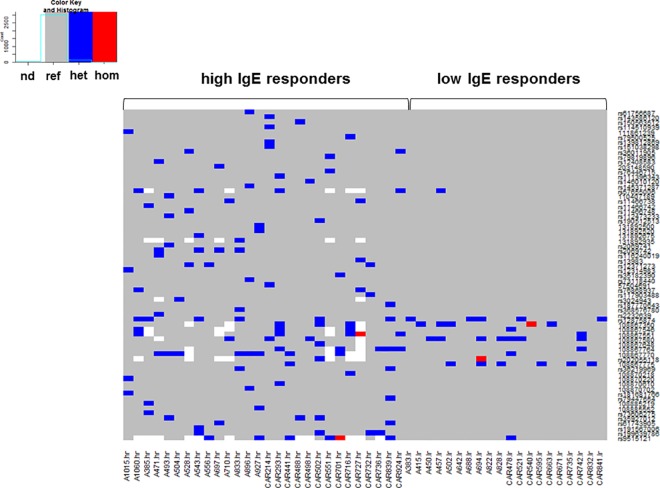
Burden of 70 single nucleotide variants with differential enrichment between high (≥75^th^ percentile) and low (≤25^th^ percentile) IgE responders to Ascaris and ABA-1. Each column corresponds to the pattern of one individual. The color scale indicates the reference genotype (grey) or the presence of single nucleotide variants in heterozygous (blue) or homozygous (red).

We analyzed 1955 variants (out of 2423) with call quality of 100 in the Phred Scale and a read depth of 30x for their enrichment according to the intensity of the IgE levels (burden analysis). Seventy variants, distributed among 8 genes (*CHIA*, *CHI3L1*, *TSLP*, *IL13*, *LIG4*, *ABHD13*, *IRS2 and STAT6*) were enriched in high IgE responders to whole *Ascaris* antigen and ABA-1 (IgE level ≥ 75^th^ percentile) and absent in low responders. These included 65 single nucleotide variations (SNVs), three insertions and two deletions, 70% of them being non-coding variants. Coding variants included 11 missense; 8 synonymous, 1 stop gain and 1 causing a frameshift change. The distribution of variants enriched in high IgE responders to *Ascaris* is shown in **Fig 1.**

### Variants associated with the IgE responses to *Ascaris* and ABA-1

**Fig 2:**
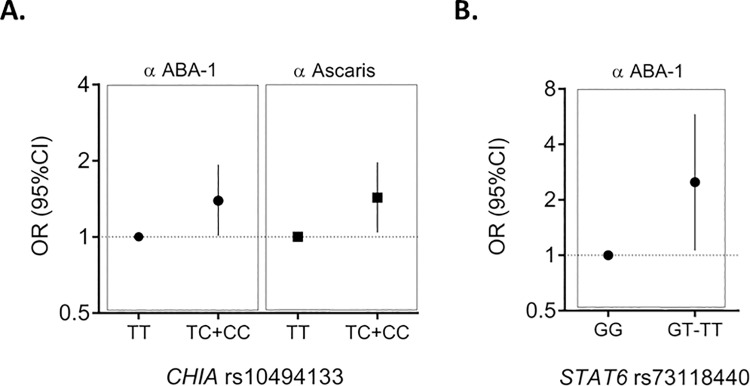
Genetic loci associated with the risk of having high IgE response (≥75^th^ percentile) to ABA-1 and *Ascaris* extract in the CGA dataset. A) Effects of the tagSNP *CHIA* rs10494133 on the risk of high IgE response to ABA-1 (filled circle) and to the *Ascaris* extract (filled square) under dominant model. B) Effects of *STAT6* rs73118440 on the risk of high IgE response to ABA-1. OR: Odds ratio; CI: confidence interval.

**Table 4** shows all the genetic associations found in this study. The burden analysis revealed two coding variants in *CHIA* (rs79500525, G/A, and rs139812869, G/A) that were enriched in high IgE responders to *Ascaris* extract and ABA-1. They were located 508 base pairs apart and in strong linkage disequilibrium (D’ = 0.99). These variants were tagged by an intronic SNP (rs10494133, T/C), observed in 14% of the individuals in the CGA dataset and associated with IgE levels to ABA-1 above percentile 75^th^ under additive (aOR: 1.39, 95%CI 1.04–1.85, p_adj_ = 0.02) and dominant models (aOR: 1.39, 95%CI 1.01–1.93, p_adj_ = 0.04), **Fig 2A**.

**Table 4:** Genetic variants associated with the strength of the IgE response in Colombian and Swedish populations

Gene	SNP	Allele	Phenotype	Population	aOR (95%CI)	model	P_adj_	P_adj_ (quantreg)^a^
*CHIA*	rs10494133	T/C	high IgE to ABA-1	Colombia	1.39 (1.04–1.85)	additive	0.02	0.04
					1.39 (1.01–1.93)	dominant	0.04	
*CHIA*	rs10494133	T/C	high IgE to *Ascaris*	Colombia	1.35 (1.02–1.79)	additive	0.02	0.2
					1.43 (1.04–1.97)	dominant	0.03	
*STAT6*	rs73118440	G/T	high IgE to ABA-1	Colombia	2.49 (1.06–5.83)	additive	0.03	0.006
*IRS2*	rs12584136	C/A	high IgE to *D*. *pteronyssinus*	Colombia	2.14 (1.24–3.67)	additive	0.007	0.03
*TNFSF13B*	rs17565502	A/C	high IgE to Fel d 1	Sweden	1.87 (1.07–3.29)	additive	0.02	0.003
*CHI3L1*	rs4950928	C/G	high IgE to ABA-1	Colombia	1.77 (1.02–3.09)	recessive	0.04	0.003
*CHI3L1*	rs4950928	C/G	high IgE to Bet v 1	Sweden	2.52 (1.21–5.25)	dominant	0.01	0.02
*CHI3L1*	rs880633	C/G	high IgE to Bet v 1	Sweden	1.82 (1.07–3.10)	additive	0.02	0.004
					2.44 (1.07–5.57)	recessive	0.03	
*ABHD13*	rs3783118	A/C	IgE to *Ascaris* below 75^th^ percentile	Colombia	0.53 (0.31–0.89)	additive	0.01	0.00001
					0.53 (0.31–0.90)	dominant	0.01	
*ABHD13*	rs3783118	A/C	IgE to *D*. *pteronyssinus* below 75^th^ percentile	Colombia	0.49 (0.28–0.87)	additive	0.01	0.13
					0.50 (0.28–0.89)	dominant	0.01	
*TNFSF13B*	rs17565502	A/C	high total IgE	Colombia	1.78 (1.16–2.75)	additive	0.009	0.03^b^
					1.75 (1.09–2.83)	dominant	0.02	
*TNFSF13B*	rs8181791	A/G	high total IgE	Sweden	1.97 (1.14–3.41)	additive	0.01	0.0006
					4.81 (1.72–13.4)	recessive	0.002	
*IRS2*	rs12584136	C/A	high total IgE	Colombia	2.71 (1.25–5.90)	allelic	0.01	0.00007^c^
*IL5*	rs2069816	A/C	high total IgE	Colombia	2.33 (1.08–5.0)	additive	0.03	0.02^b^
					2.64 (1.18–5.91)	dominant	0.02	
*IL13*	rs20541	G/A	high total IgE	Sweden	1.74 (1.0–3.0)	additive	0.05	0.008

^a^ The model fits quantile 75th (τ = 0.75) and computed the standard errors by using the Powell kernel version of the covariance matrix estimate (se =“ker”).

^b^ Estimated model on quantile 50th (τ = 0.50).

^c^ For the case of this variant the standard error was computed by the method “nid” which presumes local (in tau) linearity of the conditional quantile functions and computes a Huber sandwich estimate using a local estimate of the sparsity.

Haplotype analysis revealed a significant increased risk of high IgE levels to ABA-1 (OR: 2.32, 95%CI 1.03–5.23, p = 0.04) in carriers of the minor allele A in the two coding variants and the minor allele C in rs10494133 (global haplotype p value = 0.04 **(Table 5**).

**Table 5:** Haplotype association between genetic variants in *CHIA* and IgE response to ABA-1 in the CGA cohort (n = 988)

rs79500525	rs139812869	rs10494133	IgE to ABA-1 <75^th^ percentile (n = 736)	IgE to ABA-1 >75^th^ percentile (n = 252)	OR (95% CI)	p-value
G	G	T	0.87	0.82	1.0	-
G	G	C	0.11	0.15	1.36 (1.01–1.83)	0.04
A	A	C	0.008	0.02	2.32 (1.03–5.23)	0.04
A	A	T	0	0	-	-

Adjusted global haplotype association p-value = 0.04

We then implemented quantile regression analyses to model the effect of these genetic variants on IgE levels to ABA-1 as a continuous variable. The tagSNP rs10494133 (T/C) was associated with increased IgE levels to ABA-1 independently of age, gender or the presence of asthma (p_adj_ = 0.04). Since only one individual was homozygous for the A/A genotype in rs79500525 and rs139812869, dominant code for quantile regression (GG vs. GA + AA) was used, confirming that the allele A was significantly associated with increased IgE levels to ABA-1 (p = 0.03). The tagSNP *CHIA* rs10494133 T/C was also associated to high IgE responses to *Ascaris* under additive (aOR = 1.35, 95%CI 1.02–1.79, p_adj_ = 0.04) and dominant models (aOR = 1.43, 95%CI 1.04–1.97, p_adj_ = 0.03), driven by a higher frequency of the minor allele C in subjects with IgE levels to *Ascaris* above percentile 75^th^ **(Fig 2A)**. This was confirmed by quantile regression analysis (p = 0.01) but was not significant after adjusting by age, gender and the presence of asthma (p_adj_ = 0.2). There was also association between high IgE response to ABA-1 and *STAT6* rs73118440 (G/T) under additive model and quantile regression analysis, **Fig 2B**. Neither *CHIA* nor *STAT6* variants were associated with IgE responses to HDM or other common allergens.

### Variants associated with the IgE responses to common allergens

To address whether loci influencing IgE levels to *Ascaris* or ABA-1 were involved in the response to non-parasite allergens we explored associations with common allergens. In the CGA cohort *IRS2* rs12584136 C/A was associated with high IgE response to *D*. *pteronyssinus* (and not to parasite allergens) under additive model, dominant model and quantile regression analyses (**Table 4)**. In patients from the Swedish cohort (n = 170) the SNP rs17565502 (A/C) located in the gene encoding B cell activating factor (*TNFSF13B*) was associated with an increased risk of high IgE responses to the cat allergen Fel d 1(>4.52 ISU). This association was significant under the additive model and quantile regression analysis.

### Variants associated with IgE levels to both Ascaris and common allergens

**Fig 3:**
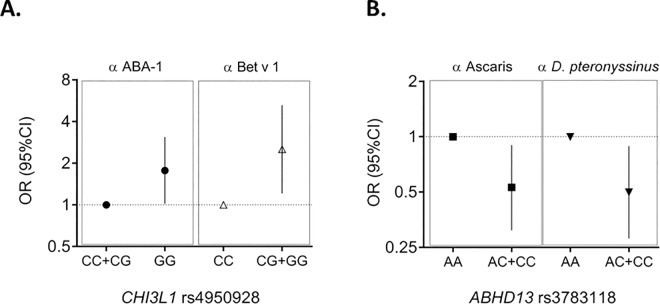
Genetic loci associated with the risk of having high IgE response to both Ascaris and common allergens. A) Effects of *CHI3L1* rs4950928 on the risk of high IgE response to ABA-1 (filled circle) and to the pollen allergen Bet v 1 (white triangle). The association with ABA-1 was detected in the CGA cohort and the association with Bet v 1 in the Swedish Eczema Cohort. **B)** Effect of *ABDH13* rs3783118 on the risk of high IgE response (≥75^th^ percentile) to the extracts of *Ascaris* and HDM in the CGA dataset. Risk of IgE response to the *Ascaris* extract (filled square); Risk of IgE response to the *D*. *pteronyssinus* extract (filled triangle). OR: Odds ratio; CI: confidence interval.

Variants in two genes were associated with both Ascaris and common allergens **(Table 4)**. In the CGA cohort *CHI3L1* (rs4950928, C/G) was associated with high IgE response to ABA-1 under recessive model, which was confirmed by quantile regression analysis, **Fig 3A**. Also, in patients from the Swedish cohort two variants in the *CHI3L1* gene (rs4950928 C/G and rs880633 C/T) were associated with high IgE response (≥75^th^ percentile) to the birch pollen allergen Bet v 1 (>10.3 ISU) **(Fig 3A)**. It is worth mentioning that these SNPs are 3081 base pairs apart, in strong LD (D’ = 0.94).

The *ABHD13* rs3783118 A/C, located in the *Ascaris* susceptibility locus (Cr. 13q33.3), was under-represented in the group of high responders to *Ascaris* under the dominant model. Quantile regression confirmed this finding showing significant association with lower IgE levels (p_adj_ = 0.00001). In the CGA dataset we found that, as occurred with the *Ascaris* extract, *ABHD13* rs3783118 was associated with lower levels of IgE to *D*. *pteronyssinus* under dominant model. This effect was driven by one or two copies of the minor allele C (p_adj_ = 0.03), **Fig 3B**.

### Variants associated with total IgE levels

**Fig 4:**
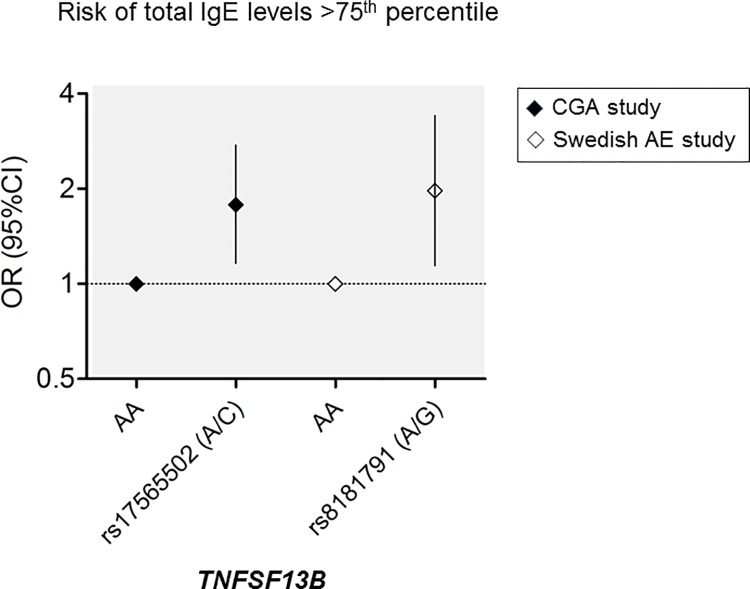
Effect *TNFSF13B* SNPs on the risk of high total serum IgE levels. Asthmatic patients from the CGA dataset (n = 391) (filled rhomboid) and AE patients from the Swedish eczema study (n = 170) (white rhomboid). OR: Odds ratio; CI: confidence interval.

**Fig 5:**
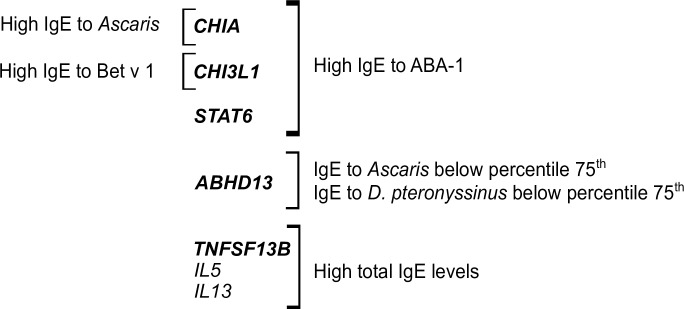
Summary of the genes influencing IgE levels in this study. Associations with those in bold are described for the first time. *TNFSF13B* was associated with total IgE levels in both Colombian and Swedish populations.

We investigate associations with high total IgE levels (≥ 75^th^ percentile) in allergic patients from the CGA dataset and the Swedish cohort. In the CGA cohort *TNFSF13B* rs17565502 A/C was associated with high total IgE levels (≥ 1074.5 IU/ml) under the additive model. In the Swedish cohort *TNFSF13B* rs8181791 A/G was associated with high total IgE levels (≥852.5 kU/l) under additive and recessive models **(Fig 4)**. Both associations were also observed when total IgE levels were modeled as a continuous variable by quantile regression **(Table 4)**. Other variants associated with high total IgE were the coding SNP *IL13* rs20541 G/A; *IRS2* rs12584136 C/A and the *IL5* rs2069816 A/C. **Fig 5** summarizes the main genetic associations found in the study.

Despite some genes evaluated in this study have been associated with asthma in particular populations, we did not find any variant significantly associated with this condition (data not shown), however, this study was designed to study QTL for total and specific IgE and is underpowered to address associations with disease phenotypes.

## Discussion

Tropical settings provide several advantages for investigating the molecular genetics of the IgE responses in helminthiases and allergies. First, due to the high prevalence of these conditions [8, 41, 42], studies analyzing the influences of genetic variants on the IgE response to allergens from both sources can be performed simultaneously in the same population; and second, the perennial exposure to allergenic components allows selecting extreme phenotypes of IgE responses. Also, it allows the comparisons with populations living in temperate climates and industrialized settings, which could be very informative. Using deep sequencing and a powerful design of extreme phenotypes case-control study [43], we discovered genetic variants in genes *CHIA* and *CHI3L1* (chromosome 1) overrepresented in high IgE responders to *Ascaris* and ABA-1 (here a purified recombinant IgE binding protein of *Ascaris*).

There is evidence for considering ABA-1 as a resistance marker for ascariasis [13], but among infected individuals some respond strongly and others not at all, despite having attested infection and immune responses to other components of the parasite [6, 13, 25]. This also occurs in animals, in association with MHC polymorphisms [7], suggesting an important genetic influence on the regulation of this response. There are no previous studies about the influence of non-MHC genes on the overall immune response to ABA-1, but we now find that genes beyond the MHC region are influential.

This is the first report of association between chitinase related genes and the IgE responsiveness to a nematode allergen (ABA-1). *CHIA* has 13 exons spanning around 29 kb and encoding AMCase, a protein with functional catalytic and chitin-binding domains in humans. This enzyme is produced by epithelial cells and alveolar macrophages, and Th2 cells are potent stimulators of its expression at both mRNA and protein level [44]. *CHI3L1* is a gene of 8 Kb, which encodes YKL40, a 40 kDa heparin and chitin-binding glycoprotein. Chitinases and chitinase-like proteins may act directly as chemotactic agents or by inducing other chemokines that attract eosinophils and T cells to the sites of parasitic infection. AMCase activity is required for the increased expression of chemokines involved in the recruitment of monocytes, macrophages, eosinophils and neutrophils. Also, it reduces the expression of the Th1 chemokines interferon gamma-inducible protein 10 (IP-10) and interferon-inducible T-cell alpha chemoattractant (I-TAC), thus contributing to a stronger Th2 response [45]. Furthermore, in mice, Ym1 (a chitinase-like protein) has been reported as a potent chemotactic agent for eosinophils and CD4^+^ T cells [46]. These functions are likely to be related to the resistance to helminths [47]; pertinently, *CHIT1* deficient individuals from South India were more susceptible to *Wuchereria bancrofti* infection [48], although such protective effect has not been replicated in other studies [49, 50]. Our study was not designed to directly investigate the genetics of susceptibility to ascariasis, but, considering the biological role of chitinases and chitinase-like proteins in the context of Th2-mediated inflammation and the predicted functional effect of the detected variants (*e*.*g*. rs880633 Arg145Gly), our findings suggest that they are relevant in the regulation of the intensity of the specific IgE response to ABA-1, which is potentially important information for understanding the genetic susceptibility to ascariasis. It is worth adding that *CHIA* rs10494133 was also associated with the intensity of the IgE response to the *Ascaris* extract, which can be explained because ABA-1 is abundant in this extract. However, the underlying mechanisms of these associations remain unknown and require functional studies.

Our results also support previous findings suggesting that *STAT6* is involved in the susceptibility to Ascaris. Gao et al [15] showed that variants of this gene were associated with low parasite infestation in humans. Therefore, our findings support indirectly the protective role of the IgE response to ABA-1. Interestingly, these genes have been associated with asthma in other populations and our results suggest that these associations could be related to their effects on IgE production, a known risk factor for asthma.

The association of *ABHD13* with the IgE responsiveness to *Ascaris* supports previous findings linking the 13q33 locus with the susceptibility to *Ascaris*-infection and the regulation of IgE responses to the parasite [18–20]. In our previous study we did not explore the effect of *ABHD13* but we now found that the rs3783118 variant was associated with lower levels of specific IgE to *Ascaris* and *D*. *pteronyssinus*. This variant generates a new binding site for the transcriptional repressor Foxp1 (Fkhd domain) that plays an important role in the differentiation of lung epithelium and is an essential transcriptional regulator of B-cell development [51]. Since there is cross reactivity between *Ascaris* and HDM extracts [14] the associations between *ABHD13* and the IgE responses to both sources may involve cross reactive components. Interestingly the SNP was not associated with the IgE response to ABA-1, which does not cross-reactive immunologically with HDM [14].

We did not include parasitological data in this study; therefore, the associations with IgE levels are not necessarily related to resistance to Ascaris infection. However, immunity to helminthiases involves a wide spectrum of effector mechanisms including the specific IgE response [52–54]. The relative importance of these antibodies has not been defined [55], although several studies have shown that elevated specific IgE levels to *Ascaris* or the purified allergen ABA-1 are associated with resistance to this nematode and decreased worm burden [13, 53, 54, 56, 57]. Since the original linkage study performed by Williams-Blangero et al. [18] identified 13q33 as a quantitative trait locus for resistance to ascariasis (as detected by parasite egg loads) and in this study we detected significant associations between genes underlying the 13q33 locus and specific IgE levels to *Ascaris*, it is feasible that genetic regulation of antibody production may play a role on *Ascaris* susceptibility.

Another aspect of the 13q33 locus is its influence on total IgE levels. In this study it was driven by variants in *TNFSF13B* present in both populations, Swedish [58] and Colombian [27]. This suggests a biological role for this gene on the regulation of total IgE levels in humans. Since high total IgE levels is a hallmark of both, helminth infections and allergic diseases, the conservative role of *TNFSF13B* on this phenotype supports potential evolutionary links between helminth immunity and allergic responses. *TNFSF13B* encodes B cell-activating factor (BAFF), a well-known major regulator of B cells development that has a critical role on the production of IgA and IgG and the synergic effect with IL-4 on the class-switching to IgE [59]. In addition, changes in BAFF levels are detectable in plasma during different immune-related conditions and nematode infections [60], and in exploring the relationship between circulating BAFF levels and antibody response in humans, we found an inverse correlation between levels of BAFF and the intensity of the antibody response to *A*. *lumbricoides* [1]. Although total IgE levels are markedly influenced by environment (mainly helminthiases), heritability of this trait was high (h^2^ = 0.53) when analyzing a large pedigree from the Jirels population in Nepal [18], which is highly exposed to *A*. *lumbricoides*. In addition, the fact that the association was also found in subjects from Sweden, where intestinal parasite infections are not endemic, rule out the potential confounding effect of helminthiases. Genomic region 13q33 is evidently of interest for further fine mapping and association studies in larger populations. Recently, another region in the chromosome 13 (13q21.31) has been suggested as locus regulating total IgE levels [61].

Another variant influencing total IgE levels in the Swedish population was *IL13* rs20541, which has been associated with allergic phenotypes and helminth susceptibility in parasite exposed populations [62–66] suggesting that this is a regulatory locus common to both allergy and parasite responses. We also found significant associations between *IL5* and total IgE levels in the Colombian population replicating previous findings on the influence of this gene on IgE production [67, 68].

Excepting *ABHD13* rs3783118, genetic variants associated with specific IgE reactivity to *Ascaris* or ABA-1 were not associated with the IgE response to HDM, suggesting that in the CGA dataset specific IgE responses to parasite and HDM allergens are controlled by different genes, which seems to contradict the idea that allergic response is a side effect of helminth immunity; however, in the Swedish cohort the IgE response against two common inhaled allergens (Bet v 1 and Fel d 1) were associated with *CHI3L1* and *TNFSF13B* respectively, which could reflect differences in the regulation of gene expression by environment. Recently, using bioinformatics tools, it was found that Bet v 1 shares an IgE binding epitope with a Bet v 1 like protein (SmBv1L) from *Schistosoma mansoni* [69]. The authors of that work confirmed the IgE binding using sera of infected individuals from Uganda, under the hypothesis that current IgE responses to common allergens is a remnant from originally protective immunity to metazoan parasites. Our findings on *CHI3L1* suggest that chitinase related genes may have evolved in the context of more primitive immune responses than those elicited by non-parasite allergens.

In summary, we have uncovered genetic variants strongly associated with the IgE response to the nematode *Ascaris* and its resistance marker ABA-1. Most associations were restricted to the response to this parasite and not to other allergens such as HDM. We also confirm previous associations, especially the relevant role of locus 13q.33 in modulating total and specific IgE levels.

## Supporting Information

S1 Fig Flow chart summarizing the research questions and phases of the study. Samples from the Colombian Dataset are indicated by the acronym CGA; Individuals were classified as high IgE responders (HR) or low IgE responders (LR) based on the percentile corresponding to their IgE levels; SKAT-O: Optimal unified Sequence Kernel Association Test; HDM: House Dust Mites; AE: atopic eczema. *Adjusted by age, gender and disease status. **For this trait only data from patients was analyzed.
(PDF)

S1 Table List of variants genotyped for the association study in CGA and the Swedish Eczema Cohort. Success rate for genotyping (%), the exact p-value for the calculations on Hardy-Weinberg equilibrium in controls (HWE) and the minor allele frequencies in each population are indicated in columns. The variants without rs number correspond to novel single nucleotide substitutions as detected in CGA.
(XLSX)

S2 Table Descriptive of sequencing metrics by gene region in the CGA cohort (DOCX)
