# Supplementary material for: Genetic Variants in CHIA and CHI3L1 Are Associated with the IgE Response to the Ascaris Resistance Marker ABA-1 and the Birch Pollen Allergen Bet v 1
Source: PLoS One. 2016 Dec 15;11(12):e0167453. doi: 10.1371/journal.pone.0167453 (PMC5157985; doi:10.1371/journal.pone.0167453)
Supplement: S1 Fig — Samples from the Colombian Dataset are indicated by the acronym CGA; Individuals were classified as high IgE responders (HR) or low IgE responders (LR) based on the percentile corresponding to their IgE levels; SKAT-O: Optimal unified Sequence Kernel Association Test; HDM: House Dust Mites; AE: atopic eczema. *Adjusted by age, gender and disease status. **For this trait only data from patients was analyzed. (PDF) [file pone.0167453.s001.pdf]

1. RESEQUENCING PHASE

Genetic variation in 14 genes	CGA dataset<br>(n = 48)	Sequencing, read mapping (hg19), variant annotation, QC + filtering → 2423 variants

2. BURDEN ANALYSIS PHASE

Are there genetic variants enriched in high IgE responders to Ascaris and ABA-1?	CGA dataset<br>(n = 48)<br><br>HR = 28 (>75th)<br>LR = 20 (< 25th)	*Ingenuity variant analysis (IVA)*<br>Confidence filter → 1955 variants<br>Predicted deletereous filter → 338 variants<br><br>Genetic and statistical associations in IVA<br>SKAT-O model for binary (HR vs. LR) and quantitative trait (IgE level to Ascaris and ABA-1)

3. ASSOCIATION STUDY PHASE

Are there variants associated with IgE response to Ascaris/ABA-1? and Are they related with the IgE response to common allergens and total IgE levels?	IgE response to Ascaris, ABA-1 and HDM	CGA dataset<br>(n=988)	*Logistic regression**<br>Association with IgE levels (above and below percentile 75th)<br><br>*Quantile regression**<br>Association with IgE levels as a continuous variable<br><br>*Mann Whitney and Kruskall Wallis tests*
	IgE response to common allergens in AE patients	Swedish AE dataset (n=170)	
	total IgE in allergic patients**	CGA dataset<br>(n = 391)<br>Swedish AE dataset<br>(n = 170)	
