# Supplementary material for: Genetic Variants in CHIA and CHI3L1 Are Associated with the IgE Response to the Ascaris Resistance Marker ABA-1 and the Birch Pollen Allergen Bet v 1
Source: PLoS One. 2016 Dec 15;11(12):e0167453. doi: 10.1371/journal.pone.0167453 (PMC5157985; doi:10.1371/journal.pone.0167453)
Supplement: S2 Table — (DOCX) [file pone.0167453.s003.docx]

| **S2 Table.** Descriptive of sequencing metrics by gene region in the CGA cohort | | | | | | | | | | | | | | |
| --- | --- | --- | --- | --- | --- | --- | --- | --- | --- | --- | --- | --- | --- | --- |
| **Chrom** | **Gene** | **# Reads** | **% Aligned** | **% duplicated** | **Insert size** | **%Reads on target** | **Mean coverage** | **% 10X cov** | **% 30X cov** | **Total variations** | **In dbSNP** | **Ts/Tv all** | **Ts/Tv (dbSNP)** | **Ts/Tv (novel)** |
| 1 | *CHIA* | 421858 | 41.80 | 32.06 | 256 | 89.9 | 736 | 100 | 99.5 | 145 | 138 | 2.54 | 2.53 | 3 |
| 1 | *IL10* | 308778 | 33.01 | 31.66 | 256 | 89.0 | 906 | 100 | 99.7 | 27 | 23 | 1.27 | 1.33 | 1 |
| 1 | *FCER1A* | 449056 | 43.20 | 32.00 | 259 | 83.2 | 636 | 100 | 98.5 | 75 | 69 | 1.80 | 1.78 | 2 |
| 1 | *CHI3L1* | 430195 | 38.73 | 31.35 | 258 | 88.0 | 871 | 100 | 99.3 | 67 | 58 | 2.14 | 2.05 | 3 |
| 5 | *TSLP* | 316097 | 36 | 31.4 | 258 | 89.3 | 687 | 100 | 98.1 | 42 | 32 | 1.16 | 1.29 | 0.8 |
| 5 | *IL13* | 279437 | 30.6 | 31.2 | 259 | 90.1 | 719 | 99 | 96.5 | 29 | 24 | 2.00 | 2.29 | 1 |
| 5 | *IL4* | 334026 | 35 | 31.7 | 257 | 86.9 | 811 | 99 | 98.7 | 46 | 42 | 2.15 | 1.92 | 3 |
| 5 | *IL5* | 272047 | 32.6 | 31.2 | 259 | 78.4 | 639 | 100 | 98.1 | 53 | 44 | 2.00 | 1.92 | 2.5 |
| 5 | *IL33* | 824546 | 56.6 | 31.9 | 254 | 85.1 | 626 | 99.8 | 97.8 | 219 | 192 | 1.41 | 1.41 | 1.5 |
| 9 | *STAT6* | 583424 | 44.2 | 31.6 | 257 | 89.2 | 787 | 100 | 98.6 | 62 | 56 | 4.18 | 4.89 | 1 |
| 12 | *LIG4* | 351454 | 38.4 | 31.6 | 256 | 91.2 | 607 | 98 | 95.6 | 101 | 76 | 1.87 | 1.95 | 1.63 |
| 13 | *ABHD13* | 390925 | 42.0 | 31.8 | 258 | 88.4 | 558 | 99.4 | 95.3 | 71 | 60 | 1.68 | 1.55 | 3 |
| 13 | *TNFSF13B* | 709635 | 55 | 31.9 | 256 | 82.8 | 547 | 99.7 | 96.1 | 205 | 172 | 1.68 | 1.68 | 1.67 |
| 13 | *IRS2* | 918602 | 57.4 | 31.6 | 259 | 91.7 | 628 | 98.5 | 94.8 | 204 | 181 | 2.07 | 2.24 | 1.11 |
| Here is shown % for coverage 10X and 30X. Ts/Tv = Transition transversion for all and separating by included in dbSNP. Ts/Tv (novel) = not included in dbSNP | | | | | | | | | | | | | | |
